# Supplementary material for: The temporal distribution of ridership in metro stations from land-use perspective
Source: PLoS One. 2024 Sep 3;19(9):e0308759. doi: 10.1371/journal.pone.0308759 (PMC11371217; doi:10.1371/journal.pone.0308759)
Supplement: Attachment — Submitted filename: Response Letter.docx [file pone.0308759.s002.docx]

**Response to Reviewers’ Comments**

Jul 10, 2024

***Journal:*** PLOS ONE

***Paper ID:*** PONE-D-24-04620

***Title of Paper:*** The Temporal Distribution of Ridership in Metro Stations from Land-use Perspective

We appreciate the time and effort of the editor and the reviewers on this manuscript. Our responses to each individual comment are listed as follows. Note that all changes accordingly are highlighted in red in the marked-up copy of the manuscript.

# **Reviewer #1:**

## *1. The aim need to specify in the abstract.*

**Response:**

Thank you for your feedback. We have revised the abstract to clearly define the aim of the study. Specifically, our goal is to explore the temporal distribution patterns of passenger flow as well as their influencing factors of land use attributes. The results can help decision-makers develop a reasonable land use development strategy around subway stations that balances utilization rates during peak and off-peak hours, thereby improving the service levels and operational efficiency of the metro system.

## *2. Line 207, the author used 800 meters, why use this value.*

**Response:**

Thank you for your query regarding the justification of the 800-meter buffer zone around subway stations. Although there is no universally mandated distance threshold for stations’ catchment areas, the 800-meter catchment area is widely recognized and supported in urban transportation research. Notably, studies such as Gan et al. (2020)^[1]^ and Liu et al. (2016)^[2]^ have utilized this buffer zone in their analyses to assess the impact of the built environment on metro ridership at both station and station-to-station level. These references provide empirical evidence supporting the appropriateness of this distance and have been duly cited and discussed in our manuscript to justify our choice.

We have already made the necessary revisions on Line 244-248 in the revised paper.

## *3. Need to clear the time period of data collection of 30 minutes intervals.*

**Response:**

Thank you for your query regarding the time period of data collection in 30-minute intervals. The metro card swiping data was supplied by the Xi'an Rail Transit Group Co., Ltd. This dataset includes processed data on boardings and alightings for 153 stations, covering the entire operational day from 4:00 AM to 00:00 AM, segmented into 30-minute intervals. We specifically used data from 19 working days in May 2021. The totaling data points is 232,560 (40 time intervals × 153 stations × 19 days × 2) accounting for both boardings and alightings. For our analysis, we calculated the average boardings and alightings for each 30-minute interval, from 4:00 AM to 00:00 AM, across these 19 working days at each station.

We have made the necessary revisions on Lines 229-237 of the revised paper.

## *4. Add ref. for land search website.*

**Response:**

Thank you for your suggestion regarding the inclusion of a reference for the land search website. We have supplemented the manuscript with references to the Landserach website. The relevant URL can be found in the footnote on Page 17 of the revised paper:

https://www.landsearch.com/blog/land-use-types-definitions

## *5. R^2^ is low for the second and third components, need to explain.*

**Response:**

Thank you for your insightful comment. The persistently low R² values for the second and third principal components indicate that land use has a limited influence on these specific aspects. The second component is designed to adjust for peak traffic during morning and evening rush hours, reflecting temporal variations in ridership. The third component captures the stochastic nature of passenger flow fluctuations, both which are less predictable and more random.

Relevant discussions are supplemented on Page 18 of the revised paper (Line 364 – Line 369).

## *6. Line 301 need to justify the writing.*

**Response:**

Thank you for your careful review. The writing on Line 301 has been fixed. We have thoroughly scanned the entire paper to ensure clarity and precision in our presentation.

## *7. Add paragraph for the work limitations and recommendation.*

**Response:**

Thank you for your valuable feedback. In response, we have added a separate subsection titled "Implications and Limitations" in the Conclusion section to address the work's limitations and provide recommendations. This subsection aims to offer a comprehensive overview of the study's scope and suggest areas for future research to enhance the findings. Please refer to this new subsection on Pages 29-30 for a detailed discussion.

# **Reviewer #2:**

## *Thanks for presenting the manuscript entitled "The Temporal Distribution of Ridership in Metro Stations from Land-use Perspective". In this study, the relationship between the land use ratio around urban rail transit stations and the temporal distribution of passenger flows is explored using methods such as principal component analysis and geographical weighted regression (GWR) models. This study provides an important reference for optimizing land use around metro stations. However, the manuscript fails to meet the publication criteria of PLOS ONE in the following key aspects:*

## *1.* *The innovativeness and research significance are not clearly articulated. Further optimization and refinement of the thesis structure and writing presentation are required.*

**Response:**

Thank you for your constructive feedback. In response, we have rewritten the introduction section to clearly specify the research gap in current studies and highlight the innovation of our work. Additionally, we have carefully edited the entire manuscript to optimize the thesis structure and ensure clarity and precision in our presentation.

Specifically, our study breaks new ground by moving beyond the traditional analysis of passenger flow features and partial segments to a comprehensive examination of the underlying temporal distribution patterns at metro stations. Our use of principal component analysis (PCA) dissects the fundamental component curves of passenger flow distributions, revealing how various land uses influence overall daily patterns. Additionally, by applying the geographically weighted regression (GWR) model, we analyze the spatial dependencies between these temporal patterns and the surrounding built environment. This dual approach uncovers the fundamental mechanisms by which land use impacts temporal distributions and offers practical insights for optimizing land use mixes to balance peak and off-peak passenger flows. Our research provides a theoretical foundation for urban planners to design more efficient and balanced metro systems, enhancing operational efficiency and passenger experience.

## *2. The experiments are too simple, and the comparative analyses are not sufficient to prove the reliability and sophistication of the experiments.*

**Response:**

Thank you for your comment regarding the experiments. Our primary objective is to understand the temporal distribution of metro ridership and identify the key land use attributes that affect it. This is an innovative study topic that, to our knowledge, has not been explored before.

In our experiments, we used PCA to identify the basic temporal distribution patterns and extract features related to metro travel behavior. Additionally, we employed both OLS and GWR models to explore the key land use attributes affecting the principal components of the temporal distribution. The GWR models also allowed us to investigate the spatial heterogeneity of these impacts.

While the methods we adopted may not appear overly complex, they are well-suited to the novel topic at hand. The experiments effectively address the research questions and provide meaningful insights. The simplicity of the methods does not detract from the study's significance; rather, it ensures clarity and robustness in understanding the interplay between land use attributes and metro ridership patterns.

## *3. Abbreviations should be given in parentheses the first time a technical term, such as PCA is used, in the manuscript the font is too small in part of line 301, the initial letter in line 305 is not capitalized, and there are grammatical errors in parts of the article.*

**Response:**

Thank you for your meticulous review and valuable feedback. We have addressed all the issues you highlighted in our manuscript:

- We have ensured that all abbreviations are clearly defined in parentheses upon their first occurrence in the text to aid understanding.
- We have adjusted the font size in Line 301 to ensure consistency and readability throughout the manuscript.
- The initial letter in Line 305 has been corrected to start with a capital letter as per standard grammatical rules.
- We have conducted a thorough review of the manuscript to correct the grammatical errors and have ensured that the language is now precise and clear.

These corrections have been made to enhance the clarity and professionalism of our manuscript.

## *4. Figures 1 to 9 in this manuscript are not clear. Please insert the original figures.*

**Response:**

Thank you for your comment regarding the clarity of the figures in the manuscript. In response, we have redrawn all the figures to ensure they are clear and accurately represent our findings. Please refer to the revised figures in the updated manuscript.

## *Overall, I cannot recommend it to be published at the current stage. Hope my comments will help authors to improve the manuscript.*

**Response:**

Thank you for your constructive feedback. We have made extensive revisions to the manuscript in response to your valuable comments. We have clarified the research objectives, enhanced the analysis, and detailed the study results along with their policy implications. We believe these improvements address the concerns raised and significantly strengthen the manuscript. We appreciate your input and hope the revised version meets the publication standards.

# **Reviewer #3:**

## *I have carefully reviewed the manuscript titled “Temporal Distribution of Ridership in Metro Stations from a Land-use Perspective.” Overall, the study provides valuable insights into the relationship between land use and passenger flow at urban rail transit stations.*

## *I have identified several major comments and questions that I believe need to be addressed before the manuscript can be considered for publication. However, I recommend addressing the following points:*

## *1. Data Collection and Representativeness:*

- *Could you provide more details about the data sources used for passenger flow analysis? How was the data collected, and what is the sample size?*

**Response:**

Thank you for your comment regarding the data sources used for passenger flow analysis. In response, we have supplemented the manuscript with detailed information about each of the data source, particularly concerning the metro card swiping data. To be more specific, the metro card swiping data was supplied by the Xi'an Rail Transit Group Co., Ltd, which includes processed data of boardings and alightings for 153 metro stations, covering the entire operational day from 4:00 AM to 00:00 AM, segmented into 30-minute intervals. We specifically used data from 19 working days in May 2021, totaling 232,560 data points. For our analysis, we calculated the average boardings and alightings for each 30-minute interval across these 19 working days at each station. These averages were then integrated into our model to ensure a comprehensive and robust dataset, enhancing the reliability and accuracy of our analytical results.

Detailed descriptions can be found on Lines 229-237 of the revised paper.

- *How representative is the dataset of the overall metro system ridership?*

**Response:**

Thank you for your question regarding the representativeness of our dataset. The dataset we used, covering 153 stations and comprising over 232,560 individual data points from 19 working days within a single month, is highly representative of the overall metro system ridership in Xi'an. This period includes a variety of typical workdays, which avoids public holidays and weekends, ensuring that the data reflects regular commuter patterns and peak usage times.

By capturing data from every station within the system and at all operational hours, the dataset not only provides a detailed snapshot of daily activity but also allows for comprehensive analysis of passenger flows, usage trends, and station-specific dynamics across the entire network. These factors contribute significantly to the reliability and generalizability of our findings to the broader context of urban metro ridership under normal conditions.

## *2. Introduction:*

- *The introduction provides a clear and concise overview of the research problem. I suggested the following articles added in the manuscript:*
- *"The analysis of utilizing multiple fences in high-speed tracks on the aerodynamic characteristics of a high-speed train model," Iranian Journal of Science and Technology, Transactions of Mechanical Engineering, 2023.*
- *"The Influence of Inclined Barriers on Airflow over a High Speed Train under Crosswind Condition, In A. G. Hessami and R. Muttram, Book: New Research on Railway Engineering and Transportation, Intech Open, 2023, ISBN 978-1-83768-620-9.*
- *"The optimum model determination of porous barriers in high-speed tracks," Journal of Rail and Rapid Transit, Vol. 236, Issue 1, pp. 15-25, 2022.*
- *"Multi Objective Optimization of Aerodynamic Design of High Speed Railway Windbreaks using Lattice Boltzmann Method and Wind Tunnel Test Results", International Journal of Rail Transportation, 2018.*
- *"2D and 3D numerical and experimental analyses of the aerodynamic effects of air fences on a high-speed train", Journal of Wind and Structures, Vol. 32, No. 6, pp.539-550, 2021.*
- *"Two-dimensional analysis of the influence of windbreaks on airflow over a high-speed train under crosswind by using Lattice Boltzmann Method ", Journal of Rail and Rapid Transit, Vol. 232, Issue. 3, pp. 863-872, 2018.*
- *"The Impact of Air Fences Geometry on Air Flow around an ICE3 High Speed Train on a Double Line Railway Track with exposure to Crosswinds", Journal of Applied Fluid Mechanics, Vol. 11, No. 3, pp. 743-754, 2018.*

**Response:**

Thank you for your valuable suggestions. We have incorporated the relevant papers into the introduction section of our manuscript.

## *3. Interpretation of Principal Components:*

- *In the PCA analysis, how were the principal components interpreted? Specifically, what practical implications do the first, second, and third components have for transit planning?*

**Response:**

Thank you for your insightful question regarding the interpretation of the principal components in our PCA analysis and their practical implications for transit planning. In our analysis, the first, second, and third principal components were interpreted as follows:

- **The first principal component** influences the peak hour ridership, reflecting the characteristics of mandatory travel patterns. This component captures the core usage patterns associated with commuter travel during rush hours.
- **The second principal component** adjusts the shape of the temporal distribution during peak hours. This modification involves refining the intensity, duration, and direction of peak period ridership, possibly addressing fluctuations within those peak times.
- **The third principal component** aims to balance the disparities between peak and off-peak periods. It functions to smooth out the distribution of ridership across the day, reducing the extremity of rush hour peaks while enhancing off-peak ridership levels.

Overall, the interpretation of these principal components provides valuable insights into passenger flow dynamics and supports the development of targeted strategies to optimize metro system operations, improve passenger experience, and enhance the efficiency of transit services.

We have discussed both the interpretation of the principal components and their practical implications in detail on Lines 317–334 of the revised paper.

- *Were any other dimensionality reduction techniques considered besides PCA?*

**Response:**

Thank you for your comment regarding dimensionality reduction techniques. While Principal Component Analysis (PCA) was chosen for its effectiveness in identifying fundamental temporal distribution patterns and extracting key features, we acknowledge that other dimensionality reduction techniques could also be considered, such as t-SNE or Factor Analysis. In this study, PCA was particularly suitable given its ability to handle the type of data we analyzed and its widespread use in similar research contexts (Luo et al. (2017)^[3]^, and Lakhina et al. (2004)^[4]^). However, future research could explore the application of alternative techniques to compare results and potentially uncover additional insights.

We have supplemented this discussion on Lines 124-130 of the revised paper.

## *4. Spatial Variation and GWR:*

- *Could you elaborate on the spatial dependence analysis using the geographically weighted regression (GWR) model? How does it account for spatial heterogeneity?*

**Response:**

Thank you for your comment regarding the spatial dependence analysis using the geographically weighted regression (GWR) model.

The GWR model enhances traditional regression analysis by allowing the relationships between variables to vary over space. Unlike the Ordinary Least Squares (OLS) model, which assumes that the relationship between dependent and independent variables is constant across the study area, the GWR model acknowledges that these relationships can change from one location to another.

In our study, the GWR model was used to analyze how land use attributes influence the principal components of temporal distribution of metro ridership at different locations. By applying GWR, different coefficients are assigned to each station, reflecting how the influence of land use attributes varies spatially. This allows us to observe local variations and identify areas where certain land uses have more or less impact on the temporal distribution of metro ridership. By accounting for spatial heterogeneity, the GWR model provides a more nuanced understanding of the factors influencing metro ridership, leading to more targeted and effective urban planning and transportation strategies.

A detailed explanation has been added to Lines 180-183 and Lines 250-257 of our revised manuscript.

- *Were there specific areas around Xi’an where the relationships between factor loadings and influencing factors differed significantly?*

**Response:**

Thank you for your question. We have addressed this topic extensively in a subsection of our manuscript titled "The Spatial Effect of Variables on the Temporal Distribution Patterns at Stations." In the revised version, we have expanded our discussions, providing deeper insights into how the relationships between factor loadings and influencing factors vary significantly across different areas of Xi'an, especially in relation to residential, educational, and commercial & office land uses. For more detailed information, please refer to Pages 22-26 of the revised manuscript.

## *5. Land Use Factors and Recommendations:*

- *Which specific land use types (e.g., residential, commercial, healthcare) contribute most significantly to morning and evening peak flows?*

**Response:**

Thank you for your query. In response to your question regarding which specific land use types contribute most significantly to morning and evening peak flows:

Residential land use is the primary contributor to shaping morning and evening peak passenger flows at metro stations. This land use generates rigid passenger flows for commuting, playing a critical role in the stability and reliability of the metro system.

Commercial & office land, along with healthcare land, play significant roles in optimizing the time distribution of passenger flows at metro stations. These land uses effectively reduce the sharpness of passenger flows during peak hours by suppressing peak flows and increasing off-peak flows, achieving a more balanced distribution of passenger flows. Constructing commercial-office complexes can boost commercial activities during off-peak periods, alleviating peak-hour pressure and increasing metro utilization during non-peak hours. Healthcare and parkland land uses attract passenger flows with dispersed time distributions, reducing peak-hour concentration.

For more detailed information, please refer to Pages 19-22 of the revised manuscript.

- *Based on your findings, what actionable recommendations can be made for optimizing land use around metro stations?*

**Response:**

Thank you for your inquiry. Several actionable recommendations can be made for optimizing land use around metro stations based on our findings:

To mitigate peak hour passenger influx and balance the daily distribution of passenger flow, we recommend implementing a mix of land uses around urban rail transit stations. Combining residential, commercial, office, educational, and recreational land uses can modify the curve of passenger flow distribution, effectively "flattening the peaks and filling the valleys" to promote balanced utilization of the subway system throughout the day. This approach aligns with the Chinese government's policy encouraging the integration of urban rail transit projects with land development along transit lines, fostering construction around station areas.

Additionally, land use and transportation strategies can be tailored to the characteristics of different urban areas, such as city centers and outlying districts. For example:

- In city centers where passenger volumes are already high, the focus should be on the rational mix of land uses to balance daily passenger flows.
- In peripheral areas, where passenger volumes are lower, initial development should prioritize residential, commercial, and educational land uses to foster essential commuting and schooling travel, thus boosting passenger flows and enhancing regional travel dynamics. Subsequent developments can gradually include other types of land uses to balance passenger flows throughout the day.

These policy recommendations aim to optimize urban development and transportation planning in a way that aligns with evolving urban dynamics and improves the overall efficiency and sustainability of public transit systems.

We have added a separate subsection titled "Implications and Limitations" in the conclusion section of our manuscript. It details specific strategies and considerations derived from our study, aimed at enhancing the effectiveness of land use planning in proximity to metro stations.

## *6. Practical Implications:*

- *Discuss how transit agencies and policymakers can apply the study’s findings to enhance metro system performance.*
- *Consider proposing specific interventions based on the research outcomes.*

**Response:**

Thank you for your valuable suggestion. Again, we have added a separate subsection titled "Implications and Limitations" in the conclusion section of our manuscript. It details specific practical implications derived from our study, aimed at enhancing the effectiveness of land use planning in proximity to metro stations.

1. Gan, Z., Yang, M., Feng, T. and Timmermans, H. J. (2020), ‘Examining the relationship between built environment and metro ridership at station-to-station level’, Transportation Research Part D: Transport and Environment, 82, 102332.
2. Liu, C., Erdogan, S., Ma, T. and Ducca, F. W. (2016), ‘How to increase rail ridership in Maryland: Direct ridership models for policy guidance’, Journal of Urban Planning and Development, 142(4), 04016017.
3. Luo, D., Cats, O., and Lint, H. V. (2017), ‘Analysis of network-wide transit passenger flows based on principal component analysis’, IEEE International Conference on Models & Technologies for Intelligent Transportation Systems. IEEE.
4. Lakhina, A., Papagiannaki, K., Crovella, M., et al. (2004), ‘Structural analysis of network traffic flows’, Acm Sigmetrics Performance Evaluation Review, 32(1): 61-72.
